# Variable Phenotypic Expression of *PAX2* Variants in Two Lithuanian Families with Kidney Disease

**DOI:** 10.3390/medicina61040597

**Published:** 2025-03-26

**Authors:** Deimante Brazdziunaite, Gabija Mazur, Marius Miglinas, Algirdas Utkus

**Affiliations:** 1 Department of Human and Medical Genetics, Institute of Biomedical Sciences, Faculty of Medicine, Vilnius University, 03101 Vilnius, Lithuania; algirdas.utkus@mf.vu.lt; 2 Centre for Medical Genetics, Vilnius University Hospital Santaros Klinikos, 08661 Vilnius, Lithuania; gabija.mazur@santa.lt; 3 Clinic of Gastroenterology, Nephro-Urology and Surgery, Faculty of Medicine, Vilnius University, 03101 Vilnius, Lithuania; marius.miglinas@santa.lt

**Keywords:** papillorenal syndrome, renal coloboma syndrome, *PAX2*-related disorder

## Abstract

*Background and Objectives*: Pathogenic variants in the *PAX2* gene have been associated with a spectrum of eye and kidney disorders, ranging from papillorenal syndrome (known as renal coloboma syndrome) to isolated nephrosis without kidney morphological anomalies (focal segmental glomerulosclerosis), inherited in an autosomal dominant manner. However, due to the growing number of reports of pathogenic variants in the *PAX2* gene, it is observed that genotype–phenotype correlation is not always consistent. We present patients from two unrelated families with *PAX2* pathogenic variants c.685C>T and c.250G>A, highlighting the diverse phenotypic expression of *PAX2*-related disorders. *Materials and Methods*: We analyzed clinical and genetic data from two families who were tested for genomic abnormalities using targeted next-generation sequencing and Sanger sequencing for segregation analysis. *Results*: In Family A, a 27-year-old male presented with chronic kidney disease stage 3, proteinuria, and multicystic kidney dysplasia diagnosed at 11 years old. An ophthalmologic examination revealed bilateral optic nerve dysplasia. In Family B, a 6-year-old female and her 4-year-old sister were clinically diagnosed with renal hypoplasia, while their 36-year-old father presented with chronic kidney disease stage 3, focal segmental glomerulosclerosis, and optic disc pits. Genetic analysis identified a heterozygous *PAX2* pathogenic variant c.685C>T, p.(Arg229*), in Family A and a heterozygous *PAX2* pathogenic variant c.250G>A, p.(Gly84Ser) in Family B. *Conclusions*: The literature and our data further support that the same *PAX2* variants may cause diverse kidney and ocular phenotypes among unrelated families and within the same family. Due to variable expressivity, a wide range of clinical manifestations of rare hereditary kidney diseases are still underdiagnosed, and a multidisciplinary approach is required to detect extrarenal signs of *PAX2*-related disorder.

## 1. Introduction

The *PAX2* (paired box gene 2) is a member of the PAX transcription factor gene family and plays a crucial role in the coordinated development of several organs during embryogenesis, including the kidneys and eyes [1]. Pathogenic variants in the *PAX2* gene have been associated with a spectrum of eye and kidney disorders, ranging from papillorenal syndrome (known as renal coloboma syndrome; OMIM: 120330) to isolated nephrosis without kidney morphological anomalies (focal segmental glomerulosclerosis; OMIM: 616002), inherited in an autosomal dominant manner.

Heterozygous variants in the *PAX2* gene were first described as the cause of the syndrome in 1995 in a family with renal hypoplasia and optic nerve colobomas [2]. To date, more than 160 unique reported DNA variants are documented in the updated database of *PAX2* gene variants (accessed on 7 February 2025: https://www.LOVD.nl/PAX2). Various studies have identified *PAX2* gene variants in both kidney and ophthalmologic disease cohorts, but the prevalence of *PAX2*-related disorder is still unknown. According to the literature, 92% of individuals with *PAX2* gene variants have kidney disorders, while 77% present with ophthalmologic diseases [3]. Kidney disorders associated with *PAX2* variants involve CAKUT, renal interstitial fibrosis, renal hypoplasia, cystic disorders, nephrotic syndrome, and urogenital cancers [4]. The most frequently described renal anomaly is hypodysplasia [5]. A review of reported cases of *PAX2*-related optic involvement summarized the most common ocular findings, which included optic nerve coloboma, optic disc excavation or pit, optic nerve hypoplasia, abnormal retinal vessels, strabismus, and retinal vessel abnormalities [6]. About one-half of patients presenting with optic nerve malformations and renal dysplasia harbor a pathogenic variant in the *PAX2* gene [7].

The *PAX2*-related disorder is highly variable. However, due to the growing number of reports of pathogenic variants in the *PAX2* gene, it is recognized that genotype–phenotype correlation is not always consistent. The inclusion of *PAX2* gene in targeted next-generation sequencing (NGS) gene panels for kidney diseases allows for the identification of disease-causing variants, even in cases where clinical diagnosis may not be accurate [8,9].

We present three individuals from two unrelated families who carry different *PAX2* gene variants to highlight the importance of *PAX2*-related disorders and the spectrum of phenotypic expression.

## 2. Materials and Methods

### 2.1. Ethical Compliance

This study was approved by the Vilnius Regional Biomedical Research Ethics Committee. Written informed consent was obtained from all patients.

### 2.2. Patients

In total, 3 patients with *PAX2* variants were analyzed out of a cohort of 112 individuals who underwent a genetic investigation for hereditary kidney diseases.

### 2.3. Clinical Evaluation

A retrospective review of clinical findings, including the age and onset of the first reported symptoms, family history, laboratory test results, kidney findings from radiologic investigations, and/or renal biopsy results, and ophthalmologic examination results was conducted. The glomerular filtration rate was estimated by correcting 24 h creatinine clearance, and chronic kidney disease (CKD) staging was based on the KDIGO 2024 Clinical Practice Guideline for the Evaluation and Management of Chronic Kidney Disease [10].

### 2.4. Genetic Investigations

#### 2.4.1. DNA Extraction

Patients’ genetic testing was performed using DNA extracted from peripheral blood samples via standard procedures using the phenol–chloroform–isoamyl alcohol extraction method.

#### 2.4.2. Next-Generation Sequencing

For Patients 1 and 2, next-generation sequencing analysis of genomic DNA was performed. Sequencing and primary steps of pre-processing raw sequencing data were performed by the subcontracting NGS provider (CeGaT GmbH, Tübingen, Germany) using the high-throughput next-generation Illumina (Illumina, Inc., San Diego, CA, USA) platform. Obtained sequencing data (FASTQ, BAM, VCF files) analysis was further performed in our laboratory using a validated in-house bioinformatic pipeline.

#### 2.4.3. Inherited Kidney Disease Gene Panel Analysis

Annotation and post-annotation of the sequencing data from the inherited kidney disease gene panel were further post-processed on-site using a validated in-house bioinformatic pipeline. Prioritization and interpretation of genome variants were performed using genomic tools and databases provided by the ANNOVAR 2020Jul08 program [11]. Variants were then classified by following the guidelines of the American College of Medical Genetics and Genomics.

#### 2.4.4. Sanger Sequencing

For the following segregation analysis, Sanger sequencing was performed using DNA samples of Patient’s 2 father and sister. DNA sequence flanking the familial variant of the *PAX2* gene was amplified using the PCR Master Mix (2X) according to the manufacturer’s protocol (Thermo Fisher Scientific, Waltham, MA, USA). Specific primers designed with Primer3web [12] for the *PAX2* gene familial variant were available upon request. Sanger sequencing was performed using the BigDye Terminator v3.1 Cycle Sequencing Kit (Applied Biosystems, Foster City, CA, USA), and automatic genetic analyzer ABI PRISM 3130xl (Applied Biosystems, USA) according to the manufacturer’s protocol. The obtained sequences were aligned with the main reference sequence of *PAX2* (NCBI: NM_000278.5).

## 3. Results

### 3.1. Clinical Characteristics

FAMILY A, PATIENT 1. A 27-year-old male was diagnosed with bilateral multicystic kidney dysplasia at 11 years old. Serum creatinine was 90 μmol/L at that time, but no information about urinalysis was available. The patient’s paternal grandmother had unspecified kidney disease and died at 28 years old.

**Figure 1:**
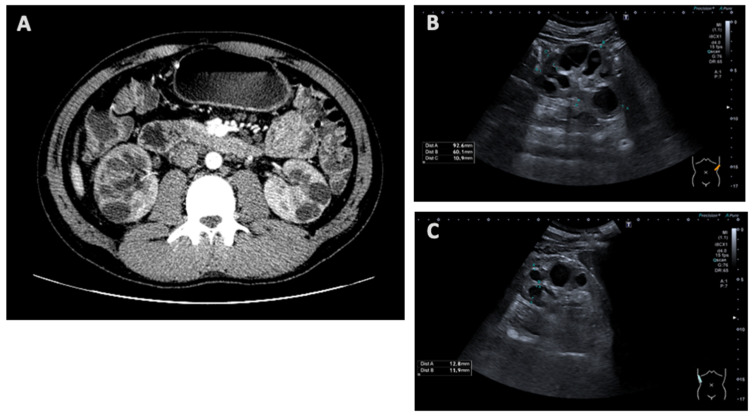
Abdominal computed tomography scan (**A**) and renal ultrasound scan (**B**,**C**) showing cystic kidneys in Patient 1.

Patient 1’s investigations were carried out to clarify the nature of his disease. The patient presented with proteinuria (1 g/L) and CKD stage G3aA3. His recent renal sonography and computed tomography showed cystic kidneys (shown in Figure 1), describing them as multicystic dysplastic or polycystic. Sonography showed that the right kidney was 96 × 48 mm and the left kidney was 100 × 49 mm in size; their parenchyma measured 14–15 mm and 15–16 mm, respectively. Both kidneys had regular contours and multiple cysts, up to 30 mm in size.

Laboratory tests revealed elevated levels of urea and creatinine and decreased estimated glomerular filtration rate (eGFR), while uric acid was within the normal range (all patients’ laboratory results are presented in Table 1). The ophthalmologic examination was performed and revealed bilateral optic nerve dysplasia without significant impact on vision. The condition remained stable over the year.

The renal function slightly decreased by the age of 30 years, and proteinuria reduced (0.3 g/L), but glucosuria was detected (up to 56 mmol/L). Kidney size remained similar to the primary sonography, and irregular contour was noted.

**Figure 2:**
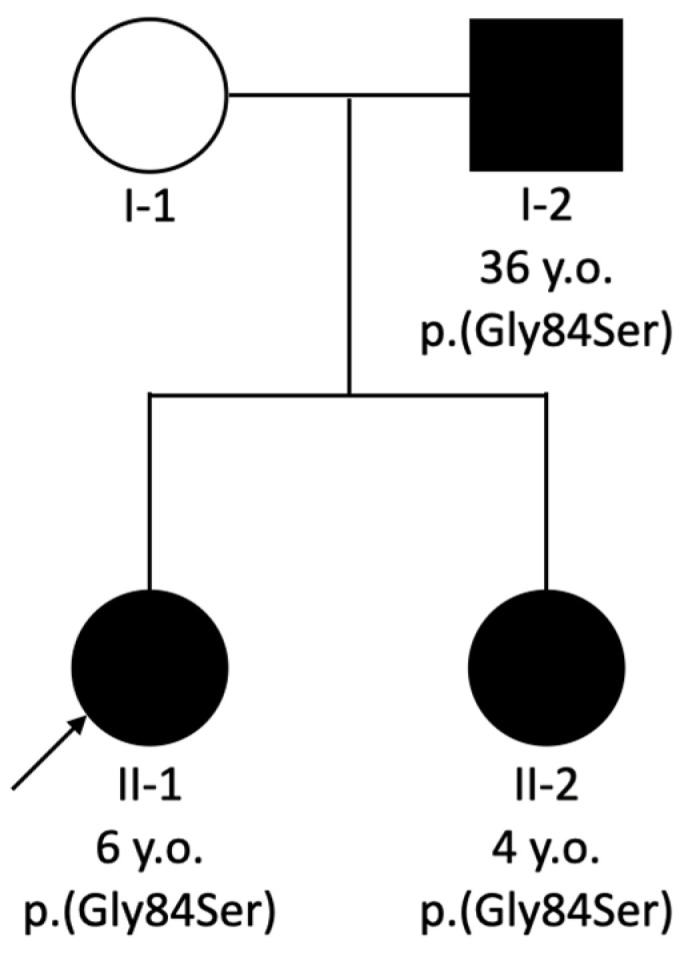
Pedigree of Family B showing *PAX2* variant segregation along with individuals’ age. The proband is marked with an arrow. Patient 2 and Patient 3 presented as II-1 and I-2, respectively.

FAMILY B, PATIENT 2. A 6-year-old female was diagnosed with bilateral renal hypoplasia shortly after birth and had arterial hypertension from two months of age. The patient’s 4-year-old sister also presented with bilateral renal hypoplasia, and their father was diagnosed with focal segmental glomerulosclerosis (FSGS) (pedigree presented in Figure 2).

At 6 years old, the patient’s growth parameters were normal. Her recent renal ultrasound scan showed signs of chronic kidney disease, microcalcifications, and bilateral simple parenchymal cysts up to 2 mm in diameter. Laboratory tests showed elevated levels of urea and creatinine and decreased eGFR; the uric acid and urinalysis results were normal.

The renal function slightly decreased by 10 years old, and the patient also developed proteinuria (0.25 g/L).

FAMILY B, PATIENT 3. The 36-year-old father of Patient 2 was referred for segregation analysis. He presented with persistent proteinuria (1 g/L) from 20 years of age and CKD stage G3bA3. Two kidney biopsies were performed since his initial diagnosis: the first one showed signs of IgA nephropathy, which was ruled out; the second one showed focal segmental glomerulosclerosis. Laboratory analysis revealed elevated levels of urea, creatinine, uric acid, and decreased eGFR. His detailed ophthalmologic examination showed optic disc pits.

The renal function slightly decreased by 39 years old, proteinuria reduced (0.3 g/L), and glucosuria was detected (up to 56 mmol/L). The renal ultrasound scan showed normal kidney size and increased parenchymal echogenicity.

### 3.2. PAX2 Variants

Next-generation sequencing of Patient 1 showed a heterozygous *PAX2* gene pathogenic variant NM_000278.5:c.685C>T, NP_000269.3:p.(Arg229*), rs76492282. Segregation analysis of this family was not possible as their parents were not available for testing.

Both Patient 2 and 3 were found to carry a heterozygous *PAX2* gene pathogenic variant NM_000278.5:c.250G>A, NP_000269.3:p.(Gly84Ser), rs2133836340, confirming the genetic cause for their kidney disease. The familial variant was also detected in the sister of Patient 2.

## 4. Discussion

Papillorenal syndrome mainly manifests by ocular and renal abnormalities due to heterozygous pathogenic *PAX2* gene variants. Both ocular and renal phenotypes present a spectrum of different findings with variable severity, even within members of the same family carrying *PAX2* variants. *PAX2* gene pathogenic variants have also been reported in individuals with isolated renal hypo/dysplasia and no ocular anomalies [13]. All this makes it difficult to determine the precise genotype–phenotype correlation of the *PAX2*-related disorder and establish diagnostic criteria.

Targeted next-generation sequencing analysis has proven to be a useful tool for both accurate etiology determination and differential diagnosis, especially in cases where patients are not necessarily extensively examined clinically before undergoing genetic testing. Domingo-Gallego et al. achieved a global diagnostic yield of 65% (300/460) in patients with early-onset CKD of suspected monogenic cause. Heterozygous variants in the *PAX2* gene were found in the majority of clinical diagnostic categories: CAKUT (three cases with dysplastic and/or hypoplastic kidneys), glomerulopathies (one case with unspecified glomerulopathy and one case with suspected Alport syndrome, accompanied by a *COL4A3* variant), and cystic kidney diseases (one case with suspected nephronophthisis-related ciliopathy and one case with unspecified polycystic kidney disease) [14]. Bullich et al. performed targeted next-generation sequencing of 140 genes associated with cystic or glomerular nephropathies in 421 patients. In this cohort, two patients carrying *PAX2* gene variants were identified: one with a clinical suspicion of nephronophthisis-related ciliopathy and the other with an unspecified glomerular kidney disease [8]. In our study, early-onset kidney disease and positive family history led to thinking of a potential underlying genetic cause. NGS was used for genetic testing, and for Patient 1 in Family A, it showed a *PAX2* gene heterozygous nonsense variant that changes the arginine at position 229 in exon 7 of the PAX2 protein homeodomain into a stop codon, leading to the production of an incomplete protein. This truncation likely disrupts the normal function of the protein and results in disease manifestation. Family B was identified with a heterozygous missense variant of *PAX2* gene, that changes G to A at position 250 in exon 2, which substitutes serine with glycine at position 84 of the protein in the highly conserved paired domain. The variant segregated with disease in this family, and its etiology was explained.

Abnormal *PAX2* gene expression disrupts kidney development, leading to various malformations. The overexpression of *PAX2* gene can inhibit nephron progenitor cell differentiation, resulting in multicystic dysplastic kidneys, which are filled with cysts and lack normal structures [15]. Conversely, insufficient *PAX2* gene expression can lead to renal hypodysplasia, characterized by underdeveloped kidneys with fewer nephrons [16]. Research and case reports on several FSGS patients revealed that *PAX2* gene variants may contribute to the familial form of FSGS, both early and adult onset [15,17], as well as lead to glomerular basement membrane changes similar to Alport syndrome [18,19].

Based on the medical literature, various mutation types were detected in *PAX2* gene, including missense, in-frame deletion, in-frame duplication, and nonsense variants [9]. A large study on 173 affected individuals seems to indicate that regardless of the type or location within the gene, heterozygous *PAX2* pathogenic variants lead to a highly penetrant and highly variable phenotype involving abnormalities of both the kidneys and optic nerve [3]. In contrast, studies with *PAX2* homozygous mutant mice demonstrated severely affected development of the optic nerve, metanephric kidney, and ventral regions of the inner ear [20], as well as early post-natal death with the absence of kidneys, ureters, and eyes [1]. Yang et al. suggested an approach predicting the pathogenic variants associated with the clinical phenotype that could be implemented in a diagnostic strategy for *PAX2*-related disorder. Their study demonstrated that renal coloboma syndrome (RCS) was highly correlated with likely/presumed gene disruptive variants (variants of deletion, frameshift, insertion, truncating, and splice site), while there were more cases with missense variants presenting with nephrosis compared to the cases with RCS, isolated congenital anomalies of the kidney and urinary tract (CAKUT), or unknown CKD [21].

To our knowledge, truncating variant p.(Arg229*) (also known as c.754C>T (p.(Arg252*)) in another transcript) detected in our study had been previously reported in a child with bilateral renal hypodysplasia, CKD stage 5, and intermittent strabismus [21]. In comparison, Patient 1 in our cohort presented with cystic kidneys diagnosed in childhood, CKD stage 3 in adulthood, and later diagnosed with bilateral optic nerve dysplasia. This contributes to the extended phenotypic spectrum associated with the same *PAX2* gene variant; however, kidney manifestation in both cases fall into the CAKUT category. The *PAX2* p.(Arg252*) genetic variant was reported in a series of severe prenatal CAKUT; a fetus with bilateral hyperechogenic kidneys, hypoplasia with cortical tubular microcysts, and focal retinal dysplasia [22]. This variant was also reported in a Chinese single-center study; however, it was not possible to match the exact clinical data of the affected individual [23].

The *PAX2* p.(Gly84Ser) pathogenic variant detected in Family B in our cohort was once described by Bower et al. in a child, who presented with nystagmus and was later found to have bilateral optic nerve colobomas with pits, but no clinically apparent renal disease in the proband or his first-degree family members carrying the same variant [3]. The concordant feature in our Patient 3 was optic disc pits, but unlike the case reported by Bower et al., the dominant feature in our Family B was renal disease, in both the child and her father. Nevertheless, our case supports Yang’s et al. suggestion that missense variants are more likely to cause nephrosis than congenital kidney anomalies.

The published medical literature and our patients’ data further support that the same *PAX2* gene variants may cause diverse kidney and ocular phenotypes among unrelated families and within the same family (as summarized in Table 2). In this study, only after establishing genetic diagnosis were patients referred to ophthalmologic assessment and were changes characteristic to *PAX2*-related disorders found. The underdiagnosis of eye lesions in patients might be attributed to the lack of awareness of *PAX2*-related disorders; the genetic diagnosis can assist in the timely detection of ocular abnormalities. On the other hand, a detailed funduscopic examination—including the optic disc—is useful for the diagnosis of kidney defects associated with *PAX2* variants [24].

Unfortunately, in the era of advancing genomic medicine, therapeutic options for *PAX2*-related disorders have not been developed yet. Li et al. suggested that silencing *PAX2* gene expression using siRNA could reduce renal tubular damage and delay interstitial fibrosis, potentially offering a new therapeutic approach for treating renal interstitial fibrosis [25]. Another study developed an induced pluripotent stem cell line from a 13-year-old boy with a heterozygous *PAX2* variant (c.226G>A, p.Gly76Ser), which could serve as a model for exploring new treatments for FSGS7 [26]. However, specific therapies for *PAX2*-related disorders remain unavailable.

## 5. Conclusions

Hereditary kidney diseases are rare and still underdiagnosed due to variable expressivity and a wide range of clinical manifestations even within single families. While some individuals with *PAX2* gene variants show mild symptoms such as isolated renal hypodysplasia, others may be affected by a more severe phenotype involving both kidney and eye abnormalities. In our patient cohort, we observed diverse phenotypes among family members and in other reported cases with the same *PAX2* pathogenic variants. Some genotype–phenotype correlations, based on mutation type, were consistent with the findings in other studies. Our study contributes to expanding the knowledge of the *PAX2*-related disorder and may contribute to a better understanding of its prevalence. A multidisciplinary approach is crucial in detecting and treating the *PAX2*-related disorder. We emphasize that an ophthalmologic assessment should be offered for individuals with renal pathology as this may lead to a more precise clinical and genetic diagnosis. Although previous studies have indicated a low overall prevalence of *PAX2* variants, the growing number of reported cases may improve our understanding of PAX2 and its role in kidney and urinary tract development and clinical presentations and provide insights for future treatment research.

## Figures and Tables

**Table 1:** Summary of laboratory test results.

	Patient 1		Patient 2		Patient 3		Reference Range
Age, Years	27	30	6	10	36	39	
Urea, mmol/L	13.5	17.2	12.38	13.7	20	20.4	2.5–7.5 (adults); 1.7–8.3 (children)
Creatinine, μmol/L	174	228	79	110	233	354	64–104 (adults); 25–42 (children)
eGFR, mL/min/1.73 m^2^	46	32	55.7	47	30	18	>90
Uric acid, μmol/L	398	338	371	476	683	379	208–428 (adults); 205–420 (children)

**Table 2:** Summary of our three cases and previously reported patients carrying corresponding *PAX2* gene variants.

	Yang et al. [21]	Madariaga et al. [22]	Family A Patient 1	Bower et al. [3]	Family B Patient 2	Family B Patient 3
Gender	F	M	M	Not specified	F	M
Age at first presentation	2.8 years	Prenatal	11 years	5.5 years	After birth	20 years
Phenotype category	CAKUT	CAKUT	CAKUT	N/A	Nephrosis	Nephrosis
Kidney imaging	N/A	Bilateral hyperechogenic kidneys	Multicystic dysplastic kidney/polycystic kidney	N/A	Signs of chronic kidney disease, microcalcifications and simple cysts	Increased parenchymal echogenicity
Kidney histology	N/A	Bilateral hypoplasia with cortical tubular microcysts	N/A	N/A	N/A	Focal segmental glomerulosclerosis
Clinical diagnosis of kidney disease	Bilateral renal hypodysplasia	N/A	Bilateral multicystic kidney dysplasia	N/A	Bilateral renal hypoplasia	Focal segmental glomerulosclerosis
Renal function	CKD stage 5 at 6 years old	N/A	CKD stage 3 at 27 years old	N/A	CKD stage 3 at 6 years old	CKD stage 3 at 36 years old
Ocular findings	Intermittent strabismus	Focal retinal dysplasia	Bilateral optic nerve dysplasia	Bilateral optic nerve coloboma; enlarged optic nerve with pits; photophobia	Normal examination	Optic disc pits
*PAX2* gene variant	c.685C>T, p.Arg229*	c.754C>T, p.Arg252*	c.685C>T, p.Arg229*	c.250G>A, p.(Gly84Ser)	c.250G>A, p.(Gly84Ser)	c.250G>A, p.(Gly84Ser)
Type of mutation	Nonsense	Nonsense	Nonsense	Missense	Missense	Missense
Zygosity	Het	Het	Het	Het	Het	Het
Segregation	N/A	Affected father	N/A	Affected father and siblings	Affected father and sibling	Affected daughters

CAKUT—congenital anomalies of the kidney and urinary tract; CKD—chronic kidney disease; Het—heterozygous.

## Data Availability

The main data generated and analyzed during this study are included in this article. Any additional information is available from the authors upon request.

## Abbreviations

The following abbreviations are used in this manuscript:

CAKUT	Congenital anomalies of the kidney and urinary tract
CKD	Chronic kidney disease
eGFR	Estimated glomerular filtration rate
FSGS	Focal segmental glomerulosclerosis
NGS	Next-generation sequencing
OMIM	Online Mendelian Inheritance in Man
RCS	Renal coloboma syndrome

## References

[1] Torres M., Gómez-Pardo E., Dressler G.R., Gruss P. (1995). *Pax-2* controls multiple steps of urogenital development. Development.

[2] Sanyanusin P., Schimmenti L.A., McNoe L.A., Ward T.A., Pierpont M.E., Sullivan M.J., Dobyns W.B., Eccles M.R. (1995). Mutation of the *PAX2* gene in a family with optic nerve colobomas, renal anomalies and vesicoureteral reflux. Nat. Genet..

[3] Bower M., Salomon R., Allanson J., Antignac C., Benedicenti F., Benetti E., Binenbaum G., Jensen U.B., Cochat P., DeCramer S. (2012). Update of *PAX2* mutations in renal coloboma syndrome and establishment of a locus-specific database. Hum. Mutat..

[4] Muntean C., Chirtes C., Baczoni B., Banescu C. (2023). *PAX2* Gene Mutation in Pediatric Renal Disorders—A Narrative Review. Int. J. Mol. Sci..

[5] Chang Y.M., Chen C.C., Lee N.C., Sung J.M., Chou Y.Y., Chiou Y.Y. (2022). *PAX2* Mutation-Related Renal Hypodysplasia: Review of the Literature and Three Case Reports. Front. Pediatr..

[6] Liu S., Zhang P., Wu J., Chang Q. (2021). A novel *PAX2* heterozygous mutation in a family with Papillorenal syndrome: A case report and review of the literature. Am. J. Ophthalmol. Case Rep..

[7] Wall P.B., Traboulsi E.I. (2013). Congenital abnormalities of the optic nerve: From gene mutation to clinical expression. Curr. Neurol. Neurosci. Rep..

[8] Bullich G., Domingo-Gallego A., Vargas I., Ruiz P., Lorente-Grandoso L., Furlano M., Fraga G., Madrid Á., Ariceta G., Borregán M. (2018). A kidney-disease gene panel allows a comprehensive genetic diagnosis of cystic and glomerular inherited kidney diseases. Kidney Int..

[9] Rossanti R., Morisada N., Nozu K., Kamei K., Horinouchi T., Yamamura T., Minamikawa S., Fujimura J., Nagano C., Sakakibara N. (2020). Clinical and genetic variability of *PAX2*-related disorder in the Japanese population. J. Hum. Genet..

[10] Kidney Disease: Improving Global Outcomes (KDIGO) CKD Work Group (2024). KDIGO 2024 Clinical Practice Guideline for the Evaluation and Management of Chronic Kidney Disease. Kidney Int..

[11] Wang K., Li M., Hakonarson H. (2010). ANNOVAR: Functional annotation of genetic variants from high-throughput sequencing data. Nucleic Acids Res..

[12] Koressaar T., Lepamets M., Kaplinski L., Raime K., Andreson R., Remm M. (2018). Primer3_masker: Integrating masking of template sequence with primer design software. Bioinformatics.

[13] Negrisolo S., Benetti E., Centi S., Della Vella M., Ghirardo G., Zanon G.F., Murer L., Artifoni L. (2011). PAX2 gene mutations in pediatric and young adult transplant recipients: Kidney and urinary tract malformations without ocular anomalies. Clin. Genet..

[14] Domingo-Gallego A., Pybus M., Bullich G., Furlano M., Ejarque-Vila L., Lorente-Grandoso L., Ruiz P., Fraga G., González M.L., Piñero-Fernández J.A. (2021). Clinical utility of genetic testing in early-onset kidney disease: Seven genes are the main players. Nephrol. Dial. Transplant..

[15] Hu X., Lin W., Luo Z., Zhong Y., Xiao X., Tang R. (2024). Frameshift Mutation in *PAX2* Related to Focal Segmental Glomerular Sclerosis: A Case Report and Literature Review. Mol. Genet. Genomic Med..

[16] Zhang L., Zhai S.B., Zhao L.Y., Zhang Y., Sun B.C., Ma Q.S. (2018). New *PAX2* heterozygous mutation in a child with chronic kidney disease: A case report and review of the literature. BMC Nephrol..

[17] Vivante A., Chacham O.S., Shril S., Schreiber R., Mane S.M., Pode-Shakked B., Soliman N.A., Koneth I., Schiffer M., Anikster Y. (2019). Dominant *PAX2* mutations may cause steroid-resistant nephrotic syndrome and FSGS in children. Pediatr. Nephrol..

[18] Ohtsubo H., Morisada N., Kaito H., Nagatani K., Nakanishi K., Iijima K. (2012). Alport-like glomerular basement membrane changes with renal-coloboma syndrome. Pediatr. Nephrol..

[19] Yamada Y., Yokoyama H., Kinoshita R., Kitamoto K., Kawaba Y., Okada S., Horie T., Nagano C., Nozu K., Namba N. (2024). Familial focal segmental glomerulosclerosis with Alport-like glomerular basement changes caused by paired box protein 2 gene variant. CEN Case Rep..

[20] Favor J., Sandulache R., Neuhäuser-Klaus A., Pretsch W., Chatterjee B., Senft E., Wurst W., Blanquet V., Grimes P., Spörle R. (1996). The mouse *Pax2*^1Neu^ mutation is identical to a human *PAX2* mutation in a family with renal-coloboma syndrome and results in developmental defects of the brain, ear, eye, and kidney. Proc. Natl. Acad. Sci. USA.

[21] Yang X., Li Y., Fang Y., Shi H., Xiang T., Liu J., Liu J., Tang X., Fang X., Chen J. (2021). Phenotypic spectrum and genetics of *PAX2*-related disorder in the Chinese cohort. BMC Med. Genom..

[22] Madariaga L., Morinière V., Jeanpierre C., Bouvier R., Loget P., Martinovic J., Dechelotte P., Leporrier N., Thauvin-Robinet C., Jensen U.B. (2013). Severe prenatal renal anomalies associated with mutations in *HNF1B* or *PAX2* genes. Clin. J. Am. Soc. Nephrol..

[23] Xiong H.Y., Shi Y.Q., Zhong C., Yang Q., Zhang G., Yang H., Wu D., Chen Y., Li Q., Wang M. (2022). Detection of De Novo *PAX2* Variants and Phenotypes in Chinese Population: A Single-Center Study. Front. Genet..

[24] Stevenson M., Pagnamenta A.T., Reichart S., Phlpott C., Lines K.E., OxClinWGS, Gorvin C.M., Lhotta K., Taylor J.C., Thakker R.V. (2020). Whole genome sequence analysis identifies a *PAX2* mutation to establish a correct diagnosis for a syndromic form of hyperuricemia. Am. J. Med. Genet. A.

[25] Li L., Wu Y., Wang C., Zhang W. (2012). Inhibition of *PAX2* Gene Expression by siRNA (Polyethylenimine) in Experimental Model of Obstructive Nephropathy. Ren. Fail..

[26] Yang X., Zhang H., Gao M., Lv Y., Song W., Duan C., Liu Y. (2023). An induced pluripotent stem cell line (SDQLCHi062-A) from a patient carrying a mutation in the *PAX2* gene. Stem Cell Res..

